# Correction: Assessing the dynamic impacts of non-pharmaceutical and pharmaceutical intervention measures on the containment results against COVID-19 in Ethiopia

**DOI:** 10.1371/journal.pone.0307327

**Published:** 2024-07-11

**Authors:** Hongli Zhu, Shiyong Liu, Wenwen Zheng, Haimanote Belay, Weiwei Zhang, Ying Qian, Yirong Wu, Tadesse Guadu Delele, Peng Jia

There are errors in the author affiliations. The correct affiliations are as follows:

Hongli Zhu^1^, Shiyong Liu^2^, Wenwen Zheng^3^, Haimanote Belay^1,4^, Weiwei Zhang^1^, Ying Qian^5^, Yirong Wu^2^, Tadesse Guadu Delele^6^, Peng Jia^7,8^

**1** Research Institute of Economics and Management, Southwestern University of Finance and Economics, Chengdu, China, **2** Institute of Advanced Studies in Humanities and Social Sciences, Beijing Normal University at Zhuhai, Zhuhai, China, **3** Personal Finance Department, HQ of China Construction Bank, Beijing, China, **4** College of Business and Economics, University of Gondar, Gondar, Ethiopia, **5** Business School, University of Shanghai for Science & Technology, Shanghai, China, **6** Department of Public Health, College of Medicine & Health Science, University of Gondar, Gondar, Ethiopia, **7** School of Resource and Environmental Sciences, Wuhan University, Wuhan, China, **8** International Institute of Spatial Lifecourse Health, Wuhan University, Wuhan, China.
