# Supplementary material for: Assessing the dynamic impacts of non-pharmaceutical and pharmaceutical intervention measures on the containment results against COVID-19 in Ethiopia
Source: PLoS One. 2022 Jul 26;17(7):e0271231. doi: 10.1371/journal.pone.0271231 (PMC9321453; doi:10.1371/journal.pone.0271231)
Supplement: S1 Appendix — (DOCX) [file pone.0271231.s001.docx]

**Appendix A**

Table A1. Estimated parameter of average contact rate during social events and routine periods

| Time | Simulation time | Social event gathering and routine life | Contact rate | Calibrated average contact rate | Sources |
| --- | --- | --- | --- | --- | --- |
| 2020/03/13-  202006/01 | 0-80 | 20-25 April 2020, more than 43% of the Ethiopia population those are Christian followers about 47 million had celebrated Easter festival in church together. (Social events) | Contact rate1 | 25.0492 | <https://www.google.com/url?q=https://www.ethiopiaid.org.uk/2020/02/an-insight-into-the-traditions-customs-and-festivities-of-fasika/> |
| 2020/06/02-08/20 | 81-160 | From June 20 to July 25, due to political conflict between parts and there were protests more than one month (high covid-19 cases). (Social events) | Contact rate2 | 21.9977 | Ethiopia: Civil Unrest - Operation Update Report 1, DREF n° MDRET022 (18 September 2020) |
| 2020/08/21-09/29 | 161-200 | Every weekend (Saturday and Sunday) almost all Christian followers have to go church for pray in mass (200-800 people in one church) and make greetings each other with kissing cheek and hand shaking which is against COvid-19 protocol. (Social events) | Contact rate3 | 21.3636 | Pew Research Center, <https://www.pewresearch.org/fact-tank/2017/11/28/ethiopia-is-an-outlier-in-the-orthodox-christian-world/> |
| 2020/09/30-2021/02/01 | 201-325 | War in between federal government of Ethiopia and one state (Tigray region) from October to December and expected more than 300000 troops involved and civilians were moved without covid-19 protocol.  During the end of December 2020, to beginning of February 2021 including x-mass, wedding month (most wedding as culture and religion direction, held in this month and in one wedding on average 300-400 people participated) and epiphany (once in a year 2000-5000 people in one place), more than 50 million people held public place event celebration. (Social events) | Contact rate4 | 29.3631 | From Wikipedia, the free encyclopedia, <https://en.wikipedia.org/wiki/Tigray_War>  Coordinating Wedding Bells - Addis <https://addisfortune.net/columns/coordinating-wedding-bells/> |
| 2021/02/02-2021/03/18 | 326-370 | During the end of December 2020, to beginning of February 2021 including x-mass, wedding month (most wedding as culture and religion direction, held in this month and in one wedding on average 300-400 people participated) and epiphany (once in a year 2000-5000 people in one place), more than 50 million people held public place event celebration. (Social events) | Contact rate5 | 27.3269 | Coordinating Wedding Bells - Addis Fortune addisfortune.net › columns › coordinating-wedding-bells, <https://addisfortune.net/columns/coordinating-wedding-bells/> |
| 2021/03/19-04/17 | 371-400 | Every weekend (Saturday and Sunday) almost all Christian followers have to go church for pray in mass (200-800 people in one church) and make greetings each other with kissing cheek and hand shaking which is against COVID-19 protocol. | Contact rate6 | 18.4591 | Pew Research Center, <https://www.pewresearch.org/fact-tank/2017/11/28/ethiopia-is-an-outlier-in-the-orthodox-christian-world/> |
| 2021/04/18-2021/06/06 | 401-450 | Every weekend (Saturday and Sunday) almost all Christian followers have to go church for pray in mass (200-800 people in one church) and make greetings each other with kissing cheek and hand shaking which is against COvid-19 protocol. | Contact rate7 | 12.1357 | Pew Research Center, <https://www.pewresearch.org/fact-tank/2017/11/28/ethiopia-is-an-outlier-in-the-orthodox-christian-world/> |
| 2021/06/07-2021/07/26 | 451-500 | Rebound of social gatherings | Contact rate8 | 20 | N/A |
| 2021/07/27-2021/09/14 | 501-550 |  | Contact rate9 | 18 | N/A |
| 2021/09/15-2021/11/05 | 501-602 | Routine and normal life | Contact rate10 | 16 | N/A |

Table A2. Death ratio without treatment*

| Real time | Simulation time | Death ratio without treatment | Calibrated value |
| --- | --- | --- | --- |
| 2020/03/13-2020/06/01 | 0-80 | death ratio without treatment1 | 0.25 |
| 2020/06/02-2020/08/20 | 81-160 | death ratio without treatment2 | 0.18 |
| 2020/08/21-2020/09/29 | 161-200 | death ratio without treatment3 | 0.15 |
| 2020/09/30-2021/02/01 | 201-325 | death ratio without treatment4 | 0.15 |
| 2021/02/02-2021/03/18 | 326-370 | death ratio without treatment5 | 0.2 |
| 2021/03/19-2021/04/17 | 371-400 | death ratio without treatment6 | 0.29994 |
| 2021/04/18-2021/06/06 | 401-450 | death ratio without treatment7 | 0.12 |
| 2021/06/07-2021/07/26 | 451-500 | death ratio without treatment8 | 0.15 |
| 2021/07/27-2021/09/14 | 501-550 | death ratio without treatment9 | 0.15 |
| 2021/09/15-2021/11/05 | 551-602 | death ratio without treatment10 | 0.395443 |

Note: * The death ratio is calculated with dividing death by severe cases.

Table A3. Death ratio with treatment*

| Real time | Simulation time | Death ratio without treatment | Calibrated value |
| --- | --- | --- | --- |
| 2020/03/13-2020/06/01 | 0-80 | death ratio with treatment1 | 0.194655 |
| 2020/06/02-2020/08/20 | 81-160 | death ratio with treatment2 | 0.15 |
| 2020/08/21-2020/09/29 | 161-200 | death ratio with treatment3 | 0.0379355 |
| 2020/09/30-2021/02/01 | 201-325 | death ratio with treatment4 | 0.0347867 |
| 2021/02/02-2021/03/18 | 326-370 | death ratio with treatment5 | 0.0981805 |
| 2021/03/19-2021/04/17 | 371-400 | death ratio with treatment6 | 0.0235328 |
| 2021/04/18-2021/06/06 | 401-450 | death ratio with treatment7 | 0.01 |
| 2021/06/07-2021/07/26 | 451-500 | death ratio with treatment8 | 0.062 |
| 2021/07/27-2021/09/14 | 501-550 | death ratio with treatment9 | 0.104628 |
| 2021/09/15-2021/11/05 | 551-602 | death ratio with treatment10 | 0.23544 |

Table A4. Calibrated symptomatic infectivity during different periods by considering social events

| Real time | Simulation time | Infectivity of symptomatic cases | Calibrated mask-wearing-adherence weighted value |
| --- | --- | --- | --- |
| 2020/03/13-2020/06/01 | 0-80 | Infectivity of symptomatic case 1 | 0.0608583 |
| 2020/06/02-2020/08/20 | 81-160 | Infectivity of symptomatic case 2 | 0.0427509 |
| 2020/08/21-2020/09/29 | 161-200 | Infectivity of symptomatic case 3 | 0.018443 |
| 2020/09/30-2021/02/01 | 201-325 | Infectivity of symptomatic case 4 | 0.0123326 |
| 2021/02/02-2021/03/18 | 326-370 | Infectivity of symptomatic case 5 | 0.0290144 |
| 2021/03/19-2021/04/17 | 371-400 | Infectivity of symptomatic case 6 | 0.0202565 |
| 2021/04/18-2021/06/06 | 401-450 | Infectivity of symptomatic case 7 | 0.0162136 |
| 2021/06/07-2021/07/26 | 451-500 | Infectivity of symptomatic case 8 | 0.01 |
| 2021/07/27-2021/09/14 | 501-550 | Infectivity of symptomatic case 9 | 0.05 |
| 2021/09/15-2021/11/05 | 551-602 | Infectivity of symptomatic case 10 | 0.0246263 |

Table A5. Calibrated asymptomatic infectivity during different periods by considering social events

| Real time | Simulation time | Infectivity of asymptomatic cases | Calibrated mask-wearing-adherence weighted value |
| --- | --- | --- | --- |
| 2020/03/13-2020/06/01 | 0-80 | Infectivity of asymptomatic case 1 | 0.00332748 |
| 2020/06/02-2020/08/20 | 81-160 | Infectivity of asymptomatic case 2 | 0.00791291 |
| 2020/08/21-2020/09/29 | 161-200 | Infectivity of asymptomatic case 3 | 0.01 |
| 2020/09/30-2021/02/01 | 201-325 | Infectivity of asymptomatic case 4 | 0.00823712 |
| 2021/02/02-2021/03/18 | 326-370 | Infectivity of asymptomatic case 5 | 0.02 |
| 2021/03/19-2021/04/17 | 371-400 | Infectivity of asymptomatic case 6 | 0.001 |
| 2021/04/18-2021/06/06 | 401-450 | Infectivity of asymptomatic case 7 | 0.00100054 |
| 2021/06/07-2021/07/26 | 451-500 | Infectivity of asymptomatic case 8 | 0.001 |
| 2021/07/27-2021/09/14 | 501-550 | Infectivity of asymptomatic case 9 | 0.01 |
| 2021/09/15-2021/11/05 | 551-602 | Infectivity of asymptomatic case 10 | 0.01 |

Table A6. Average time needed from severe symptom to recovery (treatment)

| Real time | Simulation time | Recovery period of severe symptom (with treatment) | Calibrated value |
| --- | --- | --- | --- |
| 2020/03/13-2020/08/20 | 0-160 | time from severe symptom to recovery 1 | 19.855 |
| 2020/08/20-2021/02/01 | 161-325 | time from severe symptom to recovery 2 | 13.1532 |
| 2021/02/01-2021/11/05 | 326-602 | time from severe symptom to recovery 3 | 12 |

Table A7. Average time from severe symptom to death (treatment)

| Real time | Simulation time | Survival period for severe symptom (with treatment) | Calibrated value |
| --- | --- | --- | --- |
| 2020/03/13-2020/08/20 | 0-160 | time from severe symptom to death 1 | 5.09826 |
| 2020/08/20-2021/02/01 | 161-325 | time from severe symptom to death 2 | 6.9988 |
| 2021/02/01-2021/11/05 | 326-602 | time from severe symptom to death 3 | 7.99115 |

Table A8. Recovery period from untreated severe symptom

| Real time | Simulation time | Recovery period of severe symptom (without treatment) | Calibrated value |
| --- | --- | --- | --- |
| 2020/03/13-2020/08/20 | 0-160 | recovery period from untreated severe cases1 | 22.2061 |
| 2020/08/20-2021/02/01 | 161-325 | recovery period from untreated severe cases2 | 28 |
| 2021/02/01-2021/11/05 | 326-602 | recovery period from untreated severe cases3 | 28 |

Table A9. Period from untreated severe symptom to death (untreated)

| Real time | Simulation time | Survival period for severe symptom (without treatment) | Calibrated value |
| --- | --- | --- | --- |
| 2020/03/13-2020/08/20 | 0-160 | time from untreated severe symptom to death 1 | 1 |
| 2020/08/20-2021/02/01 | 161-325 | time from untreated severe symptom to death 2 | 3 |
| 2021/02/01-2021/11/05 | 326-602 | time from untreated severe symptom to death 3 | 5 |

Table A10. Major events incorporated in the simulation of Ethiopia model

| Duration | Simulation time | The scale of social gatherings | Sources |
| --- | --- | --- | --- |
| 2020/11/04- present | 236-966  (Projected ending time) | Civil war | https://www.npr.org/2021/11/04/1052485248/rebels-are-taking-aim-at-ethiopias-capital-after-1-year-of-civil-war |
| 2022/01/07-2022 /02/07 | 665-696 | Ethiopian Christmas in Ethiopia  National Holiday (more than 70% of the population who except Muslim religion followers in Ethiopia will attend for three consecutive days. | Ethiopian Christmas in Ethiopia in 2022 \| Office Holidays  www.officeholidays.com › holidays › Ethiopia › orthodox-Christmas |
| 2022/01/17-2022/01/27 | 675-685 | Orthodox Epiphany in Ethiopia  Public holiday and only orthodox Christian 43% of population will celebrate together for ten consecutive days. | Ethiopian epiphany - intangible heritage - Culture Sector - UNESCO  ich.unesco.org › ethiopian-epiphany-01491 |

**Appendix B**

**Fig. B1 Whole stock flow diagram structure**


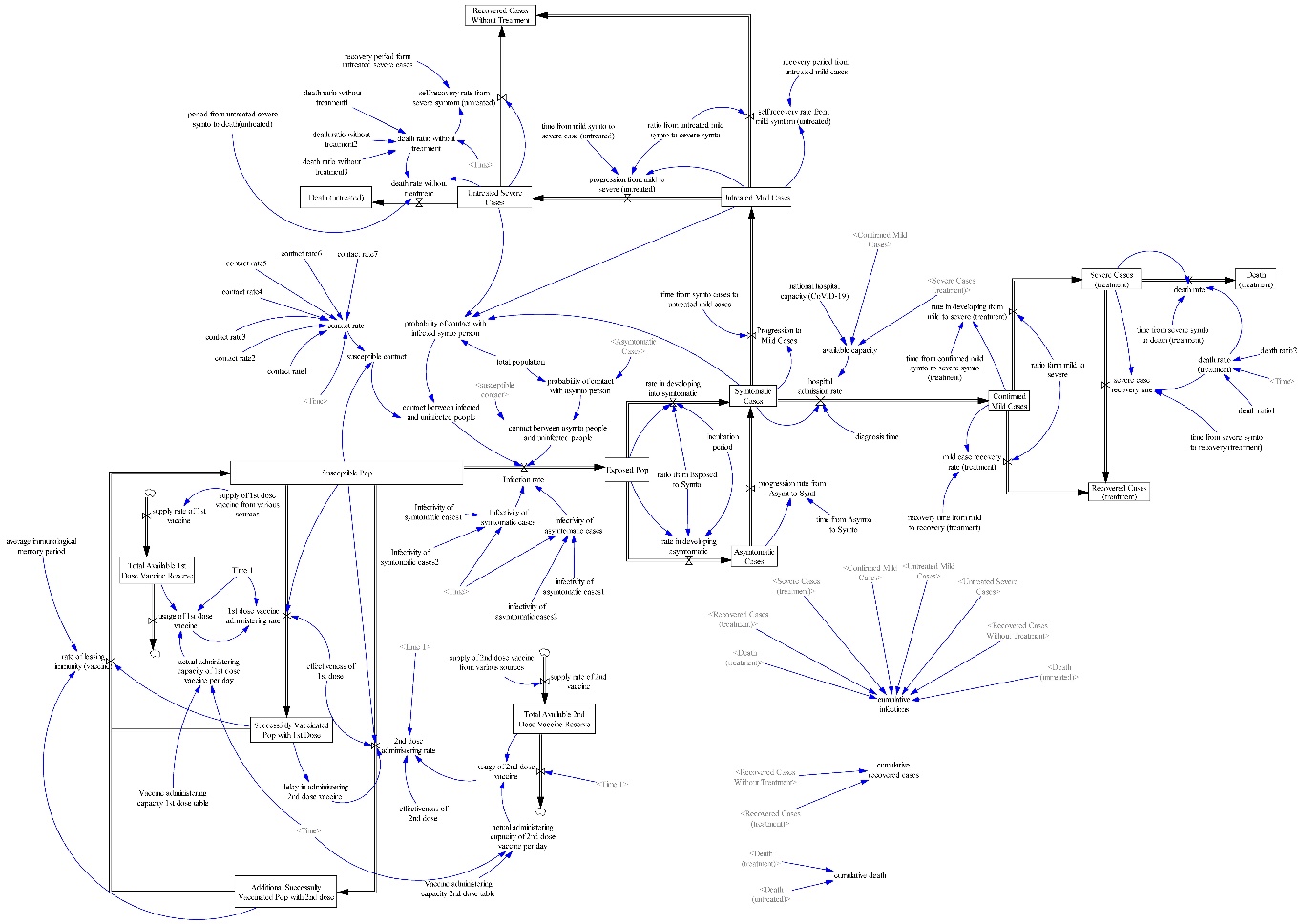


**Appendix C**

Table C1. Future scenario settings for COVID-19 transmission dynamics: S17-S64

| Scenarios | Medical resource | Vaccine supply and administration | | | | Interventions | | | |
| --- | --- | --- | --- | --- | --- | --- | --- | --- | --- |
|  | Hospital beds | Vaccine administration  (Pace calculated in historical data) | Vaccine supply  (Pace calculated in historical data) | Vaccine efficacy | Immunological period (days) | Social event-  average contact rate | Routine activities- average contact rate | Symptomatic infectivity-  weighted by mask wearing adherence | Asymptomatic infectivity-  weighted by  mask wearing adherence |
| S17 | 20000 | 10607, 8226 | 10610, 8235 | 68.4%, 80% | 240 | 24 | 16 | 48% | 48% |
| S18 | 20000 | 10607, 8226 | 10610, 8235 | 40%, 60% | 240 | 24 | 16 | 48% | 48% |
| S19 | 20000 | 10607, 8226 | 10610, 8235 | 68.4%, 80% | 180 | 24 | 16 | 48% | 48% |
| S20 | 20000 | 10607, 8226 | 10610, 8235 | 40%, 60% | 180 | 24 | 16 | 48% | 48% |
| S21 | 20000 | 10607, 8226 | 10610, 8235 | 68.4%, 80% | 240 | 22 | 14 | 48% | 48% |
| S22 | 20000 | 10607, 8226 | 10610, 8235 | 40%, 60% | 240 | 22 | 14 | 48% | 48% |
| S23 | 20000 | 10607, 8226 | 10610, 8235 | 68.4%, 80% | 180 | 22 | 14 | 48% | 48% |
| S24 | 20000 | 10607, 8226 | 10610, 8235 | 40%, 60% | 180 | 22 | 14 | 48% | 48% |
| S25 | 20000 | 10607, 8226 | 10610, 8235 | 68.4%, 80% | 240 | 20 | 12 | 48% | 48% |
| S26 | 20000 | 10607, 8226 | 10610,8235 | 40%, 60% | 240 | 20 | 12 | 48% | 48% |
| S27 | 20000 | 10607, 8226 | 10610, 8235 | 68.4%, 80% | 180 | 20 | 12 | 48% | 48% |
| S28 | 20000 | 10607, 8226 | 10610, 8235 | 40%, 60% | 180 | 20 | 12 | 48% | 48% |
| S29 | 20000 | 10607, 8226 | 10610, 8235 | 68.4%, 80% | 240 | 24 | 16 | 60% | 60% |
| S30 | 20000 | 10607, 8226 | 10610, 8235 | 40%, 60% | 240 | 24 | 16 | 60% | 60% |
| S31 | 20000 | 10607, 8226 | 10610, 8235 | 68.4%, 80% | 180 | 24 | 16 | 60% | 60% |
| S32 | 20000 | 10607, 8226 | 10610, 8235 | 40%, 60% | 180 | 24 | 16 | 60% | 60% |
| S33 | 20000 | 10607, 8226 | 10610, 8235 | 68.4%, 80% | 240 | 22 | 14 | 60% | 60% |
| S34 | 20000 | 10607, 8226 | 10610, 8235 | 40%, 60% | 240 | 22 | 14 | 60% | 60% |
| S35 | 20000 | 10607, 8226 | 10610, 8235 | 68.4%, 80% | 180 | 22 | 14 | 60% | 60% |
| S36 | 20000 | 10607, 8226 | 10610, 8235 | 40%, 60% | 180 | 22 | 14 | 60% | 60% |
| S37 | 20000 | 10607, 8226 | 10610, 8235 | 68.4%, 80% | 240 | 20 | 12 | 60% | 60% |
| S38 | 20000 | 10607, 8226 | 10610, 8235 | 40%, 60% | 240 | 20 | 12 | 60% | 60% |
| S39 | 20000 | 10607, 8226 | 10610, 8235 | 68.4%, 80% | 180 | 20 | 12 | 60% | 60% |
| S40 | 20000 | 10607, 8226 | 10610, 8235 | 40%, 60% | 180 | 20 | 12 | 60% | 60% |
| S41 | 20000 | 10607, 8226 | 10610, 8235 | 68.4%, 80% | 240 | 24 | 16 | 70% | 70% |
| S42 | 20000 | 10607, 8226 | 10610, 8235 | 40%, 60% | 240 | 24 | 16 | 70% | 70% |
| S43 | 20000 | 10607, 8226 | 10610, 8235 | 68.4%, 80% | 180 | 24 | 16 | 70% | 70% |
| S44 | 20000 | 10607, 8226 | 10610, 8235 | 40%, 60% | 180 | 24 | 16 | 70% | 70% |
| S45 | 20000 | 10607, 8226 | 10610, 8235 | 68.4%, 80% | 240 | 22 | 14 | 70% | 70% |
| S46 | 20000 | 10607, 8226 | 10610, 8235 | 40%, 60% | 240 | 22 | 14 | 70% | 70% |
| S47 | 20000 | 10607, 8226 | 10610, 8235 | 68.4%, 80% | 180 | 22 | 14 | 70% | 70% |
| S48 | 20000 | 10607, 8226 | 10610, 8235 | 40%, 60% | 180 | 22 | 14 | 70% | 70% |
| S49 | 20000 | 10607, 8226 | 10610, 8235 | 68.4%, 80% | 240 | 20 | 12 | 70% | 70% |
| S50 | 20000 | 10607, 8226 | 10610, 8235 | 40%, 60% | 240 | 20 | 12 | 70% | 70% |
| S51 | 20000 | 10607, 8226 | 10610, 8235 | 68.4%, 80% | 180 | 20 | 12 | 70% | 70% |
| S52 | 20000 | 10607, 8226 | 10610, 8235 | 40%, 60% | 180 | 20 | 12 | 70% | 70% |
| S53 | 40000 | 10607, 8226 | 10610, 8235 | 68.4%, 80% | 240 | 24 | 16 | 48% | 48% |
| S54 | 40000 | 10607, 8226 | 10610, 8235 | 40%, 60% | 240 | 24 | 16 | 48% | 48% |
| S55 | 40000 | 10607, 8226 | 10610, 8235 | 68.4%, 80% | 180 | 24 | 16 | 48% | 48% |
| S56 | 40000 | 10607, 8226 | 10610, 8235 | 40%, 60% | 180 | 24 | 16 | 48% | 48% |
| S57 | 40000 | 10607, 8226 | 10610, 8235 | 68.4%, 80% | 240 | 22 | 14 | 48% | 48% |
| S58 | 40000 | 10607, 8226 | 10610, 8235 | 40%, 60% | 240 | 22 | 14 | 48% | 48% |
| S59 | 40000 | 10607, 8226 | 10610, 8235 | 68.4%, 80% | 180 | 22 | 14 | 48% | 48% |
| S60 | 40000 | 10607, 8226 | 10610, 8235 | 40%, 60% | 180 | 22 | 14 | 48% | 48% |
| S61 | 40000 | 10607, 8226 | 10610, 8235 | 68.4%, 80% | 240 | 20 | 12 | 48% | 48% |
| S62 | 40000 | 10607, 8226 | 10610, 8235 | 40%, 60% | 240 | 20 | 12 | 48% | 48% |
| S63 | 40000 | 10607, 8226 | 10610, 8235 | 68.4%, 80% | 180 | 20 | 12 | 48% | 48% |
| S64 | 40000 | 10607, 8226 | 10610, 8235 | 40%, 60% | 180 | 20 | 12 | 48% | 48% |

Table C2. Future scenario settings for COVID-19 transmission dynamics: S64-S100

| Scenarios | Medical resource | Vaccine supply and administration | | | | Interventions | | | |
| --- | --- | --- | --- | --- | --- | --- | --- | --- | --- |
|  | Hospital beds | Vaccine administration  **^🗸🗸🗸^**（1^st^ 20%，2^nd^ 10% ） | Vaccine supply  **^🗸🗸🗸^** (1^st^ 20%，2^nd^ 10% ） | Vaccine efficacy | Immunological period (days) | Social event-  average contact rate | Routine activities- average contact rate | Symptomatic infectivity-  weighted by mask wearing adherence | Asymptomatic infectivity-  weighted by  mask wearing adherence |
| S 65 | 20000 | 55903, 25998 | 55901, 25992 | 68.4%, 80% | 240 | 24 | 16 | 48% | 48% |
| S 66 | 20000 | 55903, 25998 | 55901, 25992 | 40%, 60% | 240 | 24 | 16 | 48% | 48% |
| S 67 | 20000 | 55903, 25998 | 55901, 25992 | 68.4%, 80% | 180 | 24 | 16 | 48% | 48% |
| S 68 | 20000 | 55903, 25998 | 55901, 25992 | 40%, 60% | 180 | 24 | 16 | 48% | 48% |
| S 69 | 20000 | 55903, 25998 | 55901, 25992 | 68.4%, 80% | 240 | 22 | 14 | 48% | 48% |
| S 70 | 20000 | 55903, 25998 | 55901, 25992 | 40%, 60% | 240 | 22 | 14 | 48% | 48% |
| S 71 | 20000 | 55903, 25998 | 55901, 25992 | 68.4%, 80% | 180 | 22 | 14 | 48% | 48% |
| S 72 | 20000 | 55903, 25998 | 55901, 25992 | 40%, 60% | 180 | 22 | 14 | 48% | 48% |
| S 73 | 20000 | 55903, 25998 | 55901, 25992 | 68.4%, 80% | 240 | 20 | 12 | 48% | 48% |
| S 74 | 20000 | 55903, 25998 | 55901, 25992 | 40%, 60% | 240 | 20 | 12 | 48% | 48% |
| S 75 | 20000 | 55903, 25998 | 55901, 25992 | 68.4%, 80% | 180 | 20 | 12 | 48% | 48% |
| S 76 | 20000 | 55903, 25998 | 55901, 25992 | 40%, 60% | 180 | 20 | 12 | 48% | 48% |
| S 77 | 20000 | 55903, 25998 | 55901, 25992 | 68.4%, 80% | 240 | 24 | 16 | 60% | 60% |
| S 78 | 20000 | 55903, 25998 | 55901, 25992 | 40%, 60% | 240 | 24 | 16 | 60% | 60% |
| S 79 | 20000 | 55903, 25998 | 55901, 25992 | 68.4%, 80% | 180 | 24 | 16 | 60% | 60% |
| S 80 | 20000 | 55903, 25998 | 55901, 25992 | 40%, 60% | 180 | 24 | 16 | 60% | 60% |
| S 81 | 20000 | 55903, 25998 | 55901, 25992 | 68.4%, 80% | 240 | 22 | 14 | 60% | 60% |
| S 82 | 20000 | 55903, 25998 | 55901, 25992 | 40%, 60% | 240 | 22 | 14 | 60% | 60% |
| S 83 | 20000 | 55903, 25998 | 55901, 25992 | 68.4%, 80% | 180 | 22 | 14 | 60% | 60% |
| S 84 | 20000 | 55903, 25998 | 55901, 25992 | 40%, 60% | 180 | 22 | 14 | 60% | 60% |
| S 85 | 20000 | 55903, 25998 | 55901, 25992 | 68.4%, 80% | 240 | 20 | 12 | 60% | 60% |
| S 86 | 20000 | 55903, 25998 | 55901, 25992 | 40%, 60% | 240 | 20 | 12 | 60% | 60% |
| S 87 | 20000 | 55903, 25998 | 55901, 25992 | 68.4%, 80% | 180 | 20 | 12 | 60% | 60% |
| S 88 | 20000 | 55903, 25998 | 55901, 25992 | 40%, 60% | 180 | 20 | 12 | 60% | 60% |
| S 89 | 20000 | 55903, 25998 | 55901, 25992 | 68.4%, 80% | 240 | 24 | 16 | 70% | 70% |
| S 90 | 20000 | 55903, 25998 | 55901, 25992 | 40%, 60% | 240 | 24 | 16 | 70% | 70% |
| S 91 | 20000 | 55903, 25998 | 55901, 25992 | 68.4%, 80% | 180 | 24 | 16 | 70% | 70% |
| S 92 | 20000 | 55903, 25998 | 55901, 25992 | 40%, 60% | 180 | 24 | 16 | 70% | 70% |
| S 93 | 20000 | 55903, 25998 | 55901, 25992 | 68.4%, 80% | 240 | 22 | 14 | 70% | 70% |
| S 94 | 20000 | 55903, 25998 | 55901, 25992 | 40%, 60% | 240 | 22 | 14 | 70% | 70% |
| S 95 | 20000 | 55903, 25998 | 55901, 25992 | 68.4%, 80% | 180 | 22 | 14 | 70% | 70% |
| S 96 | 20000 | 55903, 25998 | 55901, 25992 | 40%, 60% | 180 | 22 | 14 | 70% | 70% |
| S 97 | 20000 | 55903, 25998 | 55901, 25992 | 68.4%, 80% | 240 | 20 | 12 | 70% | 70% |
| S 98 | 20000 | 55903, 25998 | 55901, 25992 | 40%, 60% | 240 | 20 | 12 | 70% | 70% |
| S 99 | 20000 | 55903, 25998 | 55901, 25992 | 68.4%, 80% | 180 | 20 | 12 | 70% | 70% |
| S 100 | 20000 | 55903, 25998 | 55901, 25992 | 40%, 60% | 180 | 20 | 12 | 70% | 70% |

Note: ^🗸🗸🗸^ it is assumed that 20% and 10% of the population in Ethiopia will be administered 1^st^ and 2^nd^ dose of COVID-19 vaccine, respectively. The number in the table represents the daily administration rate.

Table C3. Future scenario settings for COVID-19 transmission dynamics: S101-S136

| Scenarios | Medical resource | Vaccine supply and administration | | | | Interventions | | | |
| --- | --- | --- | --- | --- | --- | --- | --- | --- | --- |
|  | Hospital capacity | Vaccine administration  **^🗸🗸🗸^** (1^st^ dose 30%，2^nd^ dose 20%) | Vaccine supply  **^🗸🗸🗸^** (1^st^ dose 30%，2^nd^ dose 20%) | Vaccine efficacy | Immunological period (days) | Social event- average contact rate | Routine activities- average contact rate | Symptomatic infectivity- weighted by mask wearing adherence | Asymptomatic infectivity- weighted by mask wearing adherence |
| S 101 | 20000 | 87400, 57495 | 87398, 57489 | 68.4%, 80% | 240 | 24 | 16 | 48% | 48% |
| S 102 | 20000 | 87400, 57496 | 87398, 57490 | 40%, 60% | 240 | 24 | 16 | 48% | 48% |
| S 103 | 20000 | 87400, 57497 | 87398, 57491 | 68.4%, 80% | 180 | 24 | 16 | 48% | 48% |
| S 104 | 20000 | 87400, 57498 | 87398, 57492 | 40%, 60% | 180 | 24 | 16 | 48% | 48% |
| S 105 | 20000 | 87400, 57499 | 87398, 57493 | 68.4%, 80% | 240 | 22 | 14 | 48% | 48% |
| S 106 | 20000 | 87400, 57500 | 87398, 57494 | 40%, 60% | 240 | 22 | 14 | 48% | 48% |
| S 107 | 20000 | 87400, 57501 | 87398, 57495 | 68.4%, 80% | 180 | 22 | 14 | 48% | 48% |
| S 108 | 20000 | 87400, 57502 | 87398, 57496 | 40%, 60% | 180 | 22 | 14 | 48% | 48% |
| S 109 | 20000 | 87400, 57503 | 87398, 57497 | 68.4%, 80% | 240 | 20 | 12 | 48% | 48% |
| S 110 | 20000 | 87400, 57504 | 87398, 57498 | 40%, 60% | 240 | 20 | 12 | 48% | 48% |
| S 111 | 20000 | 87400, 57505 | 87398, 57499 | 68.4%, 80% | 180 | 20 | 12 | 48% | 48% |
| S 112 | 20000 | 87400, 57506 | 87398, 57500 | 40%, 60% | 180 | 20 | 12 | 48% | 48% |
| S 113 | 20000 | 87400, 57507 | 87398, 57501 | 68.4%, 80% | 240 | 24 | 16 | 60% | 60% |
| S 114 | 20000 | 87400, 57508 | 87398, 57502 | 40%, 60% | 240 | 24 | 16 | 60% | 60% |
| S 115 | 20000 | 87400, 57509 | 87398, 57503 | 68.4%, 80% | 180 | 24 | 16 | 60% | 60% |
| S 116 | 20000 | 87400, 57510 | 87398, 57504 | 40%, 60% | 180 | 24 | 16 | 60% | 60% |
| S 117 | 20000 | 87400, 57511 | 87398, 57505 | 68.4%, 80% | 240 | 22 | 14 | 60% | 60% |
| S 118 | 20000 | 87400, 57512 | 87398, 57506 | 40%, 60% | 240 | 22 | 14 | 60% | 60% |
| S 119 | 20000 | 87400, 57513 | 87398, 57507 | 68.4%, 80% | 180 | 22 | 14 | 60% | 60% |
| S 120 | 20000 | 87400, 57514 | 87398, 57508 | 40%, 60% | 180 | 22 | 14 | 60% | 60% |
| S 121 | 20000 | 87400, 57515 | 87398, 57509 | 68.4%, 80% | 240 | 20 | 12 | 60% | 60% |
| S 122 | 20000 | 87400, 57516 | 87398, 57510 | 40%, 60% | 240 | 20 | 12 | 60% | 60% |
| S 123 | 20000 | 87400, 57517 | 87398, 57511 | 68.4%, 80% | 180 | 20 | 12 | 60% | 60% |
| S 124 | 20000 | 87400, 57518 | 87398, 57512 | 40%, 60% | 180 | 20 | 12 | 60% | 60% |
| S 125 | 20000 | 87400, 57519 | 87398, 57513 | 68.4%, 80% | 240 | 24 | 16 | 70% | 70% |
| S 126 | 20000 | 87400, 57520 | 87398, 57514 | 40%, 60% | 240 | 24 | 16 | 70% | 70% |
| S 127 | 20000 | 87400, 57521 | 87398, 57515 | 68.4%, 80% | 180 | 24 | 16 | 70% | 70% |
| S 128 | 20000 | 87400, 57522 | 87398, 57516 | 40%, 60% | 180 | 24 | 16 | 70% | 70% |
| S 129 | 20000 | 87400, 57523 | 87398, 57517 | 68.4%, 80% | 240 | 22 | 14 | 70% | 70% |
| S 130 | 20000 | 87400, 57524 | 87398, 57518 | 40%, 60% | 240 | 22 | 14 | 70% | 70% |
| S 131 | 20000 | 87400, 57525 | 87398, 57519 | 68.4%, 80% | 180 | 22 | 14 | 70% | 70% |
| S 132 | 20000 | 87400, 57526 | 87398, 57520 | 40%, 60% | 180 | 22 | 14 | 70% | 70% |
| S 133 | 20000 | 87400, 57527 | 87398, 57521 | 68.4%, 80% | 240 | 20 | 12 | 70% | 70% |
| S 134 | 20000 | 87400, 57528 | 87398, 57522 | 68.4%, 80% | 240 | 20 | 12 | 70% | 70% |
| S 135 | 20000 | 87400, 57529 | 87398, 57523 | 68.4%, 80% | 180 | 20 | 12 | 70% | 70% |
| S 136 | 20000 | 87400, 57530 | 87398, 57524 | 40%, 60% | 180 | 20 | 12 | 70% | 70% |

Note: ^🗸🗸🗸^ it is assumed that 30% and 20% of the population in Ethiopia will be administered 1^st^ and 2^nd^ dose of COVID-19 vaccine, respectively. The number in the table represents the daily administration rate.

Table C4. Future scenario settings for COVID-19 transmission dynamics: S137-S172

| Scenarios | Medical resource | Vaccine supply and administration | | | | Interventions | | | |
| --- | --- | --- | --- | --- | --- | --- | --- | --- | --- |
|  | Hospital capacity | Vaccine administration  (1^st^ and 2^nd^ dose: pace calculated in historical data) | Vaccine supply  (1^st^ and 2^nd^ dose: pace calculated in historical data) | Vaccine efficacy | Immunological period (days) | Social event- average contact rate | Routine activities- average contact rate | Symptomatic infectivity- weighted by mask wearing adherence | Asymptomatic infectivity- weighted by mask wearing adherence |
| S 137 | 16000 | 10607, 8226 | 10610, 8235 | 68.4%, 80% | 240 | 24 | 16 | 48% | 48% |
| S 138 | 16000 | 10607, 8227 | 10610, 8236 | 40%, 60% | 240 | 24 | 16 | 48% | 48% |
| S 139 | 16000 | 10607, 8228 | 10610, 8237 | 68.4%, 80% | 180 | 24 | 16 | 48% | 48% |
| S 140 | 16000 | 10607, 8229 | 10610, 8238 | 40%, 60% | 180 | 24 | 16 | 48% | 48% |
| S 141 | 16000 | 10607, 8230 | 10610, 8239 | 68.4%, 80% | 240 | 22 | 14 | 48% | 48% |
| S 142 | 16000 | 10607, 8231 | 10610, 8240 | 40%, 60% | 240 | 22 | 14 | 48% | 48% |
| S 143 | 16000 | 10607, 8232 | 10610, 8241 | 68.4%, 80% | 180 | 22 | 14 | 48% | 48% |
| S 144 | 16000 | 10607, 8233 | 10610, 8242 | 40%, 60% | 180 | 22 | 14 | 48% | 48% |
| S 145 | 16000 | 10607, 8234 | 10610, 8243 | 68.4%, 80% | 240 | 20 | 12 | 48% | 48% |
| S 146 | 16000 | 10607, 8235 | 10610, 8244 | 40%, 60% | 240 | 20 | 12 | 48% | 48% |
| S 147 | 16000 | 10607, 8236 | 10610, 8245 | 68.4%, 80% | 180 | 20 | 12 | 48% | 48% |
| S 148 | 16000 | 10607, 8237 | 10610, 8246 | 40%, 60% | 180 | 20 | 12 | 48% | 48% |
| S 149 | 16000 | 10607, 8238 | 10610, 8247 | 68.4%, 80% | 240 | 24 | 16 | 60% | 60% |
| S 150 | 16000 | 10607, 8239 | 10610, 8248 | 40%, 60% | 240 | 24 | 16 | 60% | 60% |
| S 151 | 16000 | 10607, 8240 | 10610, 8249 | 68.4%, 80% | 180 | 24 | 16 | 60% | 60% |
| S 152 | 16000 | 10607, 8241 | 10610, 8250 | 40%, 60% | 180 | 24 | 16 | 60% | 60% |
| S 153 | 16000 | 10607, 8242 | 10610, 8251 | 68.4%, 80% | 240 | 22 | 14 | 60% | 60% |
| S 154 | 16000 | 10607, 8243 | 10610, 8252 | 40%, 60% | 240 | 22 | 14 | 60% | 60% |
| S 155 | 16000 | 10607, 8244 | 10610, 8253 | 68.4%, 80% | 180 | 22 | 14 | 60% | 60% |
| S 156 | 16000 | 10607, 8245 | 10610, 8254 | 40%, 60% | 180 | 22 | 14 | 60% | 60% |
| S 157 | 16000 | 10607, 8246 | 10610, 8255 | 68.4%, 80% | 240 | 20 | 12 | 60% | 60% |
| S 158 | 16000 | 10607, 8247 | 10610, 8256 | 40%, 60% | 240 | 20 | 12 | 60% | 60% |
| S 159 | 16000 | 10607, 8248 | 10610, 8257 | 68.4%, 80% | 180 | 20 | 12 | 60% | 60% |
| S 160 | 16000 | 10607, 8249 | 10610, 8258 | 40%, 60% | 180 | 20 | 12 | 60% | 60% |
| S 161 | 16000 | 10607, 8250 | 10610, 8259 | 68.4%, 80% | 240 | 24 | 16 | 70% | 70% |
| S 162 | 16000 | 10607, 8251 | 10610, 8260 | 40%, 60% | 240 | 24 | 16 | 70% | 70% |
| S 163 | 16000 | 10607, 8252 | 10610, 8261 | 68.4%, 80% | 180 | 24 | 16 | 70% | 70% |
| S 164 | 16000 | 10607, 8253 | 10610, 8262 | 40%, 60% | 180 | 24 | 16 | 70% | 70% |
| S 165 | 16000 | 10607, 8254 | 10610, 8263 | 68.4%, 80% | 240 | 22 | 14 | 70% | 70% |
| S 166 | 16000 | 10607, 8255 | 10610, 8264 | 40%, 60% | 240 | 22 | 14 | 70% | 70% |
| S 167 | 16000 | 10607, 8256 | 10610, 8265 | 68.4%, 80% | 180 | 22 | 14 | 70% | 70% |
| S 168 | 16000 | 10607, 8257 | 10610, 8266 | 40%, 60% | 180 | 22 | 14 | 70% | 70% |
| S 169 | 16000 | 10607, 8258 | 10610, 8267 | 68.4%, 80% | 240 | 20 | 12 | 70% | 70% |
| S 170 | 16000 | 10607, 8259 | 10610, 8268 | 40%, 60% | 240 | 20 | 12 | 70% | 70% |
| S 171 | 16000 | 10607, 8260 | 10610, 8269 | 68.4%, 80% | 180 | 20 | 12 | 70% | 70% |
| S 172 | 16000 | 10607, 8261 | 10610, 8270 | 40%, 60% | 180 | 20 | 12 | 70% | 70% |

Table C5. Future scenario settings for COVID-19 transmission dynamics: S173-S208

| Scenarios | Medical resource | Vaccine supply and administration | | | | Interventions | | | |
| --- | --- | --- | --- | --- | --- | --- | --- | --- | --- |
|  | Hospital capacity | Vaccine administration  (1^st^ and 2^nd^ dose: pace calculated in historical data) | Vaccine supply  (1^st^ and 2^nd^ dose: pace calculated in historical data) | Vaccine efficacy | Immunological period (days) | Social event- average contact rate | Routine activities- average contact rate | Symptomatic infectivity- weighted by mask wearing adherence | Asymptomatic infectivity- weighted by mask wearing adherence |
| S 173 | 12000 | 10607, 8226 | 10610, 8235 | 68.4%, 80% | 240 | 24 | 16 | 48% | 48% |
| S 174 | 12000 | 10607, 8226 | 10610, 8235 | 40%, 60% | 240 | 24 | 16 | 48% | 48% |
| S 175 | 12000 | 10607, 8226 | 10610, 8235 | 68.4%, 80% | 180 | 24 | 16 | 48% | 48% |
| S 176 | 12000 | 10607, 8226 | 10610, 8235 | 40%, 60% | 180 | 24 | 16 | 48% | 48% |
| S 177 | 12000 | 10607, 8226 | 10610, 8235 | 68.4%, 80% | 240 | 22 | 14 | 48% | 48% |
| S 178 | 12000 | 10607, 8226 | 10610, 8235 | 40%, 60% | 240 | 22 | 14 | 48% | 48% |
| S 179 | 12000 | 10607, 8226 | 10610, 8235 | 68.4%, 80% | 180 | 22 | 14 | 48% | 48% |
| S 180 | 12000 | 10607, 8226 | 10610, 8235 | 40%, 60% | 180 | 22 | 14 | 48% | 48% |
| S 181 | 12000 | 10607, 8226 | 10610, 8235 | 68.4%, 80% | 240 | 20 | 12 | 48% | 48% |
| S 182 | 12000 | 10607, 8226 | 10610, 8235 | 40%, 60% | 240 | 20 | 12 | 48% | 48% |
| S 183 | 12000 | 10607, 8226 | 10610, 8235 | 68.4%, 80% | 180 | 20 | 12 | 48% | 48% |
| S 184 | 12000 | 10607, 8226 | 10610, 8235 | 40%, 60% | 180 | 20 | 12 | 48% | 48% |
| S 185 | 12000 | 10607, 8226 | 10610, 8235 | 68.4%, 80% | 240 | 24 | 16 | 60% | 60% |
| S 186 | 12000 | 10607, 8226 | 10610, 8235 | 40%, 60% | 240 | 24 | 16 | 60% | 60% |
| S 187 | 12000 | 10607, 8226 | 10610, 8235 | 68.4%, 80% | 180 | 24 | 16 | 60% | 60% |
| S 188 | 12000 | 10607, 8226 | 10610, 8235 | 40%, 60% | 180 | 24 | 16 | 60% | 60% |
| S 189 | 12000 | 10607, 8226 | 10610, 8235 | 68.4%, 80% | 240 | 22 | 14 | 60% | 60% |
| S 190 | 12000 | 10607, 8226 | 10610, 8235 | 40%, 60% | 240 | 22 | 14 | 60% | 60% |
| S 191 | 12000 | 10607, 8226 | 10610, 8235 | 68.4%, 80% | 180 | 22 | 14 | 60% | 60% |
| S 192 | 12000 | 10607, 8226 | 10610, 8235 | 40%, 60% | 180 | 22 | 14 | 60% | 60% |
| S 193 | 12000 | 10607, 8226 | 10610, 8235 | 68.4%, 80% | 240 | 20 | 12 | 60% | 60% |
| S 194 | 12000 | 10607, 8226 | 10610, 8235 | 40%, 60% | 240 | 20 | 12 | 60% | 60% |
| S 195 | 12000 | 10607, 8226 | 10610, 8235 | 68.4%, 80% | 180 | 20 | 12 | 60% | 60% |
| S 196 | 12000 | 10607, 8226 | 10610, 8235 | 40%, 60% | 180 | 20 | 12 | 60% | 60% |
| S 197 | 12000 | 10607, 8226 | 10610, 8235 | 68.4%, 80% | 240 | 24 | 16 | 70% | 70% |
| S 198 | 12000 | 10607, 8226 | 10610, 8235 | 40%, 60% | 240 | 24 | 16 | 70% | 70% |
| S 199 | 12000 | 10607, 8226 | 10610, 8235 | 68.4%, 80% | 180 | 24 | 16 | 70% | 70% |
| S 200 | 12000 | 10607, 8226 | 10610, 8235 | 40%, 60% | 180 | 24 | 16 | 70% | 70% |
| S 201 | 12000 | 10607, 8226 | 10610, 8235 | 68.4%, 80% | 240 | 22 | 14 | 70% | 70% |
| S 202 | 12000 | 10607, 8226 | 10610, 8235 | 40%, 60% | 240 | 22 | 14 | 70% | 70% |
| S 203 | 12000 | 10607, 8226 | 10610, 8235 | 68.4%, 80% | 180 | 22 | 14 | 70% | 70% |
| S 204 | 12000 | 10607, 8226 | 10610, 8235 | 40%, 60% | 180 | 22 | 14 | 70% | 70% |
| S 205 | 12000 | 10607, 8226 | 10610, 8235 | 68.4%, 80% | 240 | 20 | 12 | 70% | 70% |
| S 206 | 12000 | 10607, 8226 | 10610, 8235 | 40%, 60% | 240 | 20 | 12 | 70% | 70% |
| S 207 | 12000 | 10607, 8226 | 10610, 8235 | 68.4%, 80% | 180 | 20 | 12 | 70% | 70% |
| S 208 | 12000 | 10607, 8226 | 10610, 8235 | 40%, 60% | 180 | 20 | 12 | 70% | 70% |

Table C6. Future scenario settings for COVID-19 transmission dynamics: S209-S244

| Scenarios | Medical resource | Vaccine supply and administration | | | | Interventions | | | |
| --- | --- | --- | --- | --- | --- | --- | --- | --- | --- |
|  | Hospital capacity | Vaccine administration  **^🗸🗸🗸^**（1^st^ 20%，2^nd^ 10% ） | Vaccine supply  **^🗸🗸🗸^** (1^st^ 20%，2^nd^ 10% ） | Vaccine efficacy | Immunological period (days) | Social event- average contact rate | Routine activities- average contact rate | Symptomatic infectivity- weighted by mask wearing adherence | Asymptomatic infectivity- weighted by mask wearing adherence |
| Scenario 209 | 16000 | 55903, 25998 | 55901, 25992 | 68.4%, 80% | 240 | 24 | 16 | 48% | 48% |
| Scenario 210 | 16000 | 55903, 25999 | 55901, 25993 | 40%, 60% | 240 | 24 | 16 | 48% | 48% |
| Scenario 211 | 16000 | 55903, 26000 | 55901, 25994 | 68.4%, 80% | 180 | 24 | 16 | 48% | 48% |
| Scenario 212 | 16000 | 55903, 26001 | 55901, 25995 | 40%, 60% | 180 | 24 | 16 | 48% | 48% |
| Scenario 213 | 16000 | 55903, 26002 | 55901, 25996 | 68.4%, 80% | 240 | 22 | 14 | 48% | 48% |
| Scenario 214 | 16000 | 55903, 26003 | 55901, 25997 | 40%, 60% | 240 | 22 | 14 | 48% | 48% |
| Scenario 215 | 16000 | 55903, 26004 | 55901, 25998 | 68.4%, 80% | 180 | 22 | 14 | 48% | 48% |
| Scenario 216 | 16000 | 55903, 26005 | 55901, 25999 | 40%, 60% | 180 | 22 | 14 | 48% | 48% |
| Scenario 217 | 16000 | 55903, 26006 | 55901, 26000 | 68.4%, 80% | 240 | 20 | 12 | 48% | 48% |
| Scenario 218 | 16000 | 55903, 26007 | 55901, 26001 | 40%, 60% | 240 | 20 | 12 | 48% | 48% |
| Scenario 219 | 16000 | 55903, 26008 | 55901, 26002 | 68.4%, 80% | 180 | 20 | 12 | 48% | 48% |
| Scenario 220 | 16000 | 55903, 26009 | 55901, 26003 | 40%, 60% | 180 | 20 | 12 | 48% | 48% |
| Scenario 221 | 16000 | 55903, 26010 | 55901, 26004 | 68.4%, 80% | 240 | 24 | 16 | 60% | 60% |
| Scenario 222 | 16000 | 55903, 26011 | 55901, 26005 | 40%, 60% | 240 | 24 | 16 | 60% | 60% |
| Scenario 223 | 16000 | 55903, 26012 | 55901, 26006 | 68.4%, 80% | 180 | 24 | 16 | 60% | 60% |
| Scenario 224 | 16000 | 55903, 26013 | 55901, 26007 | 40%, 60% | 180 | 24 | 16 | 60% | 60% |
| Scenario 225 | 16000 | 55903, 26014 | 55901, 26008 | 68.4%, 80% | 240 | 22 | 14 | 60% | 60% |
| Scenario 226 | 16000 | 55903, 26015 | 55901, 26009 | 40%, 60% | 240 | 22 | 14 | 60% | 60% |
| Scenario 227 | 16000 | 55903, 26016 | 55901, 26010 | 68.4%, 80% | 180 | 22 | 14 | 60% | 60% |
| Scenario 228 | 16000 | 55903, 26017 | 55901, 26011 | 40%, 60% | 180 | 22 | 14 | 60% | 60% |
| Scenario 229 | 16000 | 55903, 26018 | 55901, 26012 | 68.4%, 80% | 240 | 20 | 12 | 60% | 60% |
| Scenario 230 | 16000 | 55903, 26019 | 55901, 26013 | 40%, 60% | 240 | 20 | 12 | 60% | 60% |
| Scenario 231 | 16000 | 55903, 26020 | 55901, 26014 | 68.4%, 80% | 180 | 20 | 12 | 60% | 60% |
| Scenario 232 | 16000 | 55903, 26021 | 55901, 26015 | 40%, 60% | 180 | 20 | 12 | 60% | 60% |
| Scenario 233 | 16000 | 55903, 26022 | 55901, 26016 | 68.4%, 80% | 240 | 24 | 16 | 70% | 70% |
| Scenario 234 | 16000 | 55903, 26023 | 55901, 26017 | 40%, 60% | 240 | 24 | 16 | 70% | 70% |
| Scenario 235 | 16000 | 55903, 26024 | 55901, 26018 | 68.4%, 80% | 180 | 24 | 16 | 70% | 70% |
| Scenario 236 | 16000 | 55903, 26025 | 55901, 26019 | 40%, 60% | 180 | 24 | 16 | 70% | 70% |
| Scenario 237 | 16000 | 55903, 26026 | 55901, 26020 | 68.4%, 80% | 240 | 22 | 14 | 70% | 70% |
| Scenario 238 | 16000 | 55903, 26027 | 55901, 26021 | 40%, 60% | 240 | 22 | 14 | 70% | 70% |
| Scenario 239 | 16000 | 55903, 26028 | 55901, 26022 | 68.4%, 80% | 180 | 22 | 14 | 70% | 70% |
| Scenario 240 | 16000 | 55903, 26029 | 55901, 26023 | 40%, 60% | 180 | 22 | 14 | 70% | 70% |
| Scenario 241 | 16000 | 55903, 26030 | 55901, 26024 | 68.4%, 80% | 240 | 20 | 12 | 70% | 70% |
| Scenario 242 | 16000 | 55903, 26031 | 55901, 26025 | 40%, 60% | 240 | 20 | 12 | 70% | 70% |
| Scenario 243 | 16000 | 55903, 26032 | 55901, 26026 | 68.4%, 80% | 180 | 20 | 12 | 70% | 70% |
| Scenario 244 | 16000 | 55903, 26033 | 55901, 26027 | 40%, 60% | 180 | 20 | 12 | 70% | 70% |

Note: ^🗸🗸🗸^ it is assumed that 20% and 10% of the population in Ethiopia will be administered 1^st^ and 2^nd^ dose of COVID-19 vaccine, respectively. The number in the table represents the daily administration rate.

Table C7. Future scenario settings for COVID-19 transmission dynamics: S245-S280

| Scenarios | Medical resource | Vaccine supply and administration | | | | Interventions | | | |
| --- | --- | --- | --- | --- | --- | --- | --- | --- | --- |
|  | Hospital capacity | Vaccine administration  **^🗸🗸🗸^**（1^st^ 20%，2^nd^ 10% ） | Vaccine supply  **^🗸🗸🗸^** (1^st^ 20%，2^nd^ 10% ） | Vaccine efficacy | Immunological period (days) | Social event- average contact rate | Routine activities- average contact rate | Symptomatic infectivity- weighted by mask wearing adherence | Asymptomatic infectivity- weighted by mask wearing adherence |
| S 245 | 12000 | 55903, 25998 | 55901, 25992 | 68.4%, 80% | 240 | 24 | 16 | 48% | 48% |
| S 246 | 16000 | 55903, 25999 | 55901, 25993 | 40%, 60% | 240 | 24 | 16 | 48% | 48% |
| S 247 | 16000 | 55903, 26000 | 55901, 25994 | 68.4%, 80% | 180 | 24 | 16 | 48% | 48% |
| S 248 | 16000 | 55903, 26001 | 55901, 25995 | 40%, 60% | 180 | 24 | 16 | 48% | 48% |
| S 249 | 16000 | 55903, 26002 | 55901, 25996 | 68.4%, 80% | 240 | 22 | 14 | 48% | 48% |
| S 250 | 16000 | 55903, 26003 | 55901, 25997 | 40%, 60% | 240 | 22 | 14 | 48% | 48% |
| S 251 | 16000 | 55903, 26004 | 55901, 25998 | 68.4%, 80% | 180 | 22 | 14 | 48% | 48% |
| S 252 | 16000 | 55903, 26005 | 55901, 25999 | 40%, 60% | 180 | 22 | 14 | 48% | 48% |
| S 253 | 16000 | 55903, 26006 | 55901, 26000 | 68.4%, 80% | 240 | 20 | 12 | 48% | 48% |
| S 254 | 16000 | 55903, 26007 | 55901, 26001 | 40%, 60% | 240 | 20 | 12 | 48% | 48% |
| S 255 | 16000 | 55903, 26008 | 55901, 26002 | 68.4%, 80% | 180 | 20 | 12 | 48% | 48% |
| S 256 | 16000 | 55903, 26009 | 55901, 26003 | 40%, 60% | 180 | 20 | 12 | 48% | 48% |
| S 257 | 16000 | 55903, 26010 | 55901, 26004 | 68.4%, 80% | 240 | 24 | 16 | 60% | 60% |
| S 258 | 16000 | 55903, 26011 | 55901, 26005 | 40%, 60% | 240 | 24 | 16 | 60% | 60% |
| S 259 | 16000 | 55903, 26012 | 55901, 26006 | 68.4%, 80% | 180 | 24 | 16 | 60% | 60% |
| S 260 | 16000 | 55903, 26013 | 55901, 26007 | 40%, 60% | 180 | 24 | 16 | 60% | 60% |
| S 261 | 16000 | 55903, 26014 | 55901, 26008 | 68.4%, 80% | 240 | 22 | 14 | 60% | 60% |
| S 262 | 16000 | 55903, 26015 | 55901, 26009 | 40%, 60% | 240 | 22 | 14 | 60% | 60% |
| S 263 | 16000 | 55903, 26016 | 55901, 26010 | 68.4%, 80% | 180 | 22 | 14 | 60% | 60% |
| S 264 | 16000 | 55903, 26017 | 55901, 26011 | 40%, 60% | 180 | 22 | 14 | 60% | 60% |
| S 265 | 16000 | 55903, 26018 | 55901, 26012 | 68.4%, 80% | 240 | 20 | 12 | 60% | 60% |
| S 266 | 16000 | 55903, 26019 | 55901, 26013 | 40%, 60% | 240 | 20 | 12 | 60% | 60% |
| S 267 | 16000 | 55903, 26020 | 55901, 26014 | 68.4%, 80% | 180 | 20 | 12 | 60% | 60% |
| S 268 | 16000 | 55903, 26021 | 55901, 26015 | 40%, 60% | 180 | 20 | 12 | 60% | 60% |
| S 269 | 16000 | 55903, 26022 | 55901, 26016 | 68.4%, 80% | 240 | 24 | 16 | 70% | 70% |
| S 270 | 16000 | 55903, 26023 | 55901, 26017 | 40%, 60% | 240 | 24 | 16 | 70% | 70% |
| S 271 | 16000 | 55903, 26024 | 55901, 26018 | 68.4%, 80% | 180 | 24 | 16 | 70% | 70% |
| S 272 | 16000 | 55903, 26025 | 55901, 26019 | 40%, 60% | 180 | 24 | 16 | 70% | 70% |
| S 273 | 16000 | 55903, 26026 | 55901, 26020 | 68.4%, 80% | 240 | 22 | 14 | 70% | 70% |
| S 274 | 16000 | 55903, 26027 | 55901, 26021 | 40%, 60% | 240 | 22 | 14 | 70% | 70% |
| S 275 | 16000 | 55903, 26028 | 55901, 26022 | 68.4%, 80% | 180 | 22 | 14 | 70% | 70% |
| S 276 | 16000 | 55903, 26029 | 55901, 26023 | 40%, 60% | 180 | 22 | 14 | 70% | 70% |
| S 277 | 16000 | 55903, 26030 | 55901, 26024 | 68.4%, 80% | 240 | 20 | 12 | 70% | 70% |
| S 278 | 16000 | 55903, 26031 | 55901, 26025 | 40%, 60% | 240 | 20 | 12 | 70% | 70% |
| S 279 | 16000 | 55903, 26032 | 55901, 26026 | 68.4%, 80% | 180 | 20 | 12 | 70% | 70% |
| S 280 | 16000 | 55903, 26033 | 55901, 26027 | 40%, 60% | 180 | 20 | 12 | 70% | 70% |

Note: ^🗸🗸🗸^ it is assumed that 20% and 10% of the population in Ethiopia will be administered 1^st^ and 2^nd^ dose of COVID-19 vaccine, respectively. The number in the table represents the daily administration rate
