# Supplementary material for: Assessing the dynamic impacts of non-pharmaceutical and pharmaceutical intervention measures on the containment results against COVID-19 in Ethiopia
Source: PLoS One. 2022 Jul 26;17(7):e0271231. doi: 10.1371/journal.pone.0271231 (PMC9321453; doi:10.1371/journal.pone.0271231)
Supplement: Attachment — Submitted filename: Comments to authors.pdf [file pone.0271231.s003.pdf]

Comments to Authors

Currently, COVID-19 is a major public health issue and assessing the dynamic impacts of non-pharmaceutical and pharmaceutical intervention measures against COVID-19 is an important area of study. The authors try to investigate system dynamics model to capture COVID-19 characteristics, major social events, stringencies of containment measures, and vaccine dynamics. To improve the quality of the paper, I suggest to address the following issues:

Abstract

- Avoid abbreviation from the abstract. i.e. SD, SDM

Background

- The background section didn't intensively explore literatures related with this study.
- Many statements written without citing references that support the statement. Such us line 64-66, line 72-78, Line
- This section didn't motivate why doing this study is important and what are the limitation of other similar studies, i.e. Ejigu et al (2021)
- Need proof reading. There are many grammatical issues across the paper.
  i.e., line 61 "only were 1.4%....".
  line 161 "This study is to better understand…"

Method

- Line 161 is not a meaningful sentence. "This study is to better understand…"
- Motivation to the considered "Model structure and formulations" were not clearly stated. Under what assumption this model is valid? How you get the model parameter input parameters?
- Add links/hyperlinks (cite websites) from where you got the data under the "Data collection and quality" subsection.
- Further in this section, you need to explain
  a) How the Immunological period (days) 240 and 180 determined.
  b) How the vaccine efficacy determined
  c) Briefly explain how other parameters also fixed
-

Result and discussion

- Many of the assumptions set for the scenarios are far away the reality. For example, line 272-273, it was stated that ".. adherence level in wearing face was assumed 55%" which is really far away from the reality in the context of Ethiopia. As far as I know, 75% of the population in Ethiopia are living in rural areas and majority of them didn't her about facemask. Further, in line 295 "…. adherence level of mask-wearing (48%, 60%, and 70%)."
- Revise the assumed adherence level related with face masking, social distancing,… in the context of Ethiopia.
- Line 297 "…total of 968 days", but in line 205 "… total of 603 days" which one is correct.
- Finding of other studies related with this work were not well discussed.
- Limitation of the study not clearly stated. i.e., the quality of the data and others not clearly addressed.
